# Caregiving, Lack of Companionship, and Mental Health Self-Care During COVID-19

**DOI:** 10.1093/geroni/igaf122.734

**Published:** 2025-12-31

**Authors:** Niying Li, Darshan Chudasama, Lin-Na Chou, Mary Louise Pomeroy, Yiqing Qian

**Affiliations:** University of Georgia, Athens, Georgia, United States; University of Georgia College of Public Health, Athens, Georgia, United States; University of Utah, Saratoga Springs, Utah, United States; Johns Hopkins Bloomberg School of Public Health, Baltimore, Maryland, United States; Johns Hopkins University School of Nursing, Baltimore, Maryland, United States

## Abstract

The COVID-19 pandemic exposed public health challenges in social isolation and loneliness and urgent demands for mental health self-care services (e.g., professional services, wellness apps). Family caregivers face unique barriers to socialization and mental health self-care. Using nationally representative data from the 2021 National Poll on Healthy Aging of adults aged 50-80, we examined the association between a lack of companionship and use of mental health self-care services between March 2020 and January 2021, and explored family caregiver status as an effect modifier. The survey-weighted sample was mostly non-Hispanic White (70.9%), between ages 50-64 (59.5%), living alone (78.6%), and non-caregivers (87.5%). Compared with non-caregivers, caregivers were more likely to report lacking companionship (45.4% vs 35.9%) and using self-care services (46.1% vs 35.8%). In the full sample including both caregivers and non-caregivers, we conducted a logistic regression adjusting for socio-demographic and health characteristics, demonstrating that lacking companionship was associated with a higher average predicted probability of using self-care services (40.0%, 95%CI: 36.1%-44.0%) than not lacking companionship (35.9%, 95%CI: 32.9%-38.8%). Caregiver status moderated this association. Among caregivers, those lacking companionship (35.6%, 95%CI: 26.3%-45.0%) had a lower average predicted probability of self-care use than those not lacking companionship (45.8%, 95%CI: 37.3%-54.4%). Our work highlights that caregivers who lack companionship are also less likely to use mental health self-care services, suggesting a potential lack of time, resources, or knowledge to access mental health self-care during COVID-19. Future work should examine loneliness during caregiving and tailoring self-care services to caregivers’ social and emotional needs.

